# Accelerating eucalypt clone selection pipeline via cloned progeny trials and molecular data

**DOI:** 10.1186/s13007-025-01342-3

**Published:** 2025-02-14

**Authors:** Thiago Romanos Benatti, Filipe Manoel Ferreira, Rodolfo Manoel Lemes da Costa, Mario Luiz Teixeira de Moraes, Aurélio Mendes Aguiar, Donizete da Costa Dias, José Wilacildo de Matos, Aline Cristina Miranda Fernandes, Mateus Chagas Andrade, Leandro de Siqueira, Itaraju Junior Baracuhy Brum, André Vieira do Nascimento, Yuri Tani Utsunomiya, José Fernando Garcia, Evandro Vagner Tambarussi

**Affiliations:** 1 Suzano S.A, Jacareí, São Paulo Brazil; 2 Universidade Estadual Paulista “Júlio de Mesquita Filho”, Botucatu, Brazil; 3 Universidade Estadual Paulista “Júlio de Mesquita Filho”, Ilha Solteira, SP Brazil; 4 Universidade Estadual Paulista “Júlio de Mesquita Filho”, Jaboticabal, SP Brazil; 5 AgroPartners Consulting, Araçatuba, SP Brazil; 6 Universidade Estadual Paulista “Júlio de Mesquita Filho”, Araçatuba, SP Brazil

## Abstract

The high productivity of *Eucalyptus* spp. forest plantations is mainly due to advances in silvicultural techniques and genetic improvement associated with the potential that many species of the genus have for vegetative propagation. However, long reproduction cycles for forest species pose significant challenges for genetic progress via traditional breeding programs. Furthermore, there is often poor correlation between individual (seedling) performance in initial (progeny trials) and final (clonal trials) stages of the breeding program. In this scenario, cloned progeny trials (CPT) offer an alternative to accelerate the eucalypt clone selection pipeline, combining progeny and clonal trials in a single experiment. CPT has the potential to speed up the evaluation process and increase its efficiency by developing new commercial genotypes that were tested as clones from the initial stage of the breeding program. Thus, this study aims to assess the potential of CPT to accelerate eucalypt clone selection programs by estimating the genetic parameters, analyzing responses to selection, and predicting the adequate number of ramets to be used in CPT of *Eucalyptus urophylla* x *Eucalyptus grandis*. The results show that when the number of ramets per progeny was decreased from five to one there was a reduction in the estimates of broad-sense heritability and accuracy. However, three ramets/progeny can be used without significant reductions in these estimates. CPT accelerates clonal selection by combining progeny and clonal trial methodologies, enabling an evaluation of performance as both progeny and clone. This capacity is very important for vegetatively propagated crop species such as *Eucalyptus*. Integrating CPT with SNP markers can offer an alternative to shorten the tree clone selection pipeline, better estimate and decompose the genetic variance components, and improve the correlation between initial and final performance for selected genotypes. This study confirms the potential of CPT to improve selection processes and accelerate genetic gains in the eucalypt clone selection pipeline.

**Supplementary Information:**

The online version contains supplementary material available at 10.1186/s13007-025-01342-3.

## Introduction

Clonal eucalypt forests are highly uniform and productive because they utilize the best genotypes from vegetatively propagated ramets [1]. This boost in productivity occurs because cloning enables breeders to capitalize on both additive and non-additive genetic effects [2]. Consequently, cloning produces more homogeneous raw material, offering significant advantages for industry, such as reduction of production costs and standardization of the final product [3]. To develop a superior clone, the genetic variability explored in the progenies are generated via open pollination (where only the maternal parent is known) or controlled crosses of known parents (full sibs). These progenies are then evaluated in progeny tests to assess genotype performance.

In Brazil, early selection for growth traits, such as wood volume, is typically performed at approximately three years of age (half the six-year rotation cycle for eucalypts), due to a strong correlation between growth traits at this early age and the final harvest age [4]. Genotypes selected for their genetic merit undergo another cycle of crossing for further advancement and are vegetatively propagated to assess performance in clonal trials across several locations and environments [5]. Even with early selection at three years, the full clonal selection pipeline starting from recombination all the way to commercial clone recommendation can take 13 to 15 years, and up to 18 years if genotypes are slow to flower [6]. Another challenge is that the performance of a genotype (ortet or original plant) in progeny trials does not always predict (or reflect) the performance of its clones (ramets or clones of an ortet) in clonal trials [7, 8]. It is important to emphasize that there is a significant difference in root architecture of true seed seedlings compared to vegetatively propagated ramets, which can also influence an individual´s performance during progeny and clonal trial stages.

Differences among independent ramets of the same ortet can arise from physiological and morphological factors, and may be influenced by several variables, including ontogenetic stage, shoot collection position, or environmental conditions [9, 10]. These factors, which are not accounted for during progeny trials, may explain the poor correlation in yield performance between the same genotype in progeny and clonal tests [11]. This limited correlation has been observed for various forest species [12–14] and can have an impact on the efficiency of selection of superior genotypes for commercial deployment. One approach to address this issue is to independently clone genotypes of a progeny that originated from true seeds when they are young seedlings [15].

Controlled crosses are performed between top performing parents resulting in many progenies. In this case, each progeny (or family) is composed of many genotypes originating from different seeds. Therefore, each genotype will be an ortet that provides ramets via vegetative propagation to be tested in the hybrid progeny trial (HPT). This ensures that ramets remain at the same juvenile stage as their ortets, reducing the effects of ontogenetic age in cloning [11, 15]. However, by selecting a single genotype in hybrid progeny trials and cloning it, breeders overlook the fact that some traits present limited or intermediate genetic control and can be strongly influenced by the environment. Thus, breeders may underestimate the risk that ramets of a selected genotype, based on a single repetition of the phenotype, might not maintain its performance in future stages of the breeding program or in commercial plantations. Therefore, testing new strategies for eucalypt clone selection, such as cloned progeny trials (CPT) [12, 15–17], is crucial to improve consistency in performance of selected genotypes between initial and final stages [18].

CPT offer a valuable approach to integrate both progeny and clonal trials within a single experiment [19, 20]. This design allows breeders to select the best genotypes based on repetitions of the same individual. In contrast, traditional progeny trials rely on a single observation. CPT offers notable advantages: it eliminates the need to rescue and collect shoots from older progeny test candidates to establish separate clonal trials; it reduces the time required to select commercial clones; and CPT enhances the accuracy of predicted genetic effects [18]. Furthermore, establishing CPT across different environments can provide valuable insights into genotype-by-environment interactions. However, a significant limitation of this approach is that experimental setups become increasingly complex and resource intensive as the number of genotypes and replicates (complete or incomplete blocks) increases. Also, the individualization and multiplication of each progeny can be complex, making it vulnerable to genotype mixing. To mitigate this, methods such as the use of plant barcode systems from the nursery to field tests [21] and molecular markers to genotype and genetically identify CPT individuals [22] can be employed to ensure accuracy. Using molecular markers can also enable pedigree reconstruction which allows for the identification of genealogical relationships among individuals, improving precision in estimating genetic parameters caused by the bias of information about the male parent in open-pollinated orchards [23, 24].

As CPT is re-introduced into a new era where DNA and digital information is within reach, both phenotype and genotype data are becoming more reliable. The combination of CPT with genomic tools supports more precise differentiation of additive and non-additive genetic effects contributing to an understanding of a trait’s genetic architecture [25] and providing more reliable breeding values for forest tree species [26]. In addition, the use of molecular markers allows for the identification of genetic variants associated with desirable traits [27] and has the potential to improve the correlation between initial and final performance for selected genotypes due to a reduction in pedigree errors and better assessment of genetic diversity [28]. Therefore, genetic markers provide more information, that may facilitate the early selection of potential new parents and clones in CPT. In this context, this study investigates the use of CPT in multi-environment trials integrated with SNP molecular markers to select superior eucalypt clones. To do so, the objectives of this study were to: (i) assess the viability of an eucalypt clonal breeding program based on the use of CPT; (ii) estimate genetic parameters from CPT data by exploring additive and non-additive genetic effects, and estimate the rate of change in response to early selection for the 30 best-ranked genotypes; and (iii) predict the effect of different numbers of ramets/ortet on estimates of heritability and accuracy. To the best of our knowledge, no other study has developed CPT of subtropical *Eucalyptus* species in a multi-environment scenario associated with molecular data. Our findings shed light onto how CPT can be used to improve selection efficiency and indicate the minimum number of ramets/ortet necessary for accurate selection in eucalypt breeding programs.

## Materials and methods

### Breeding population and cloned progeny trial

**Fig. 1:**
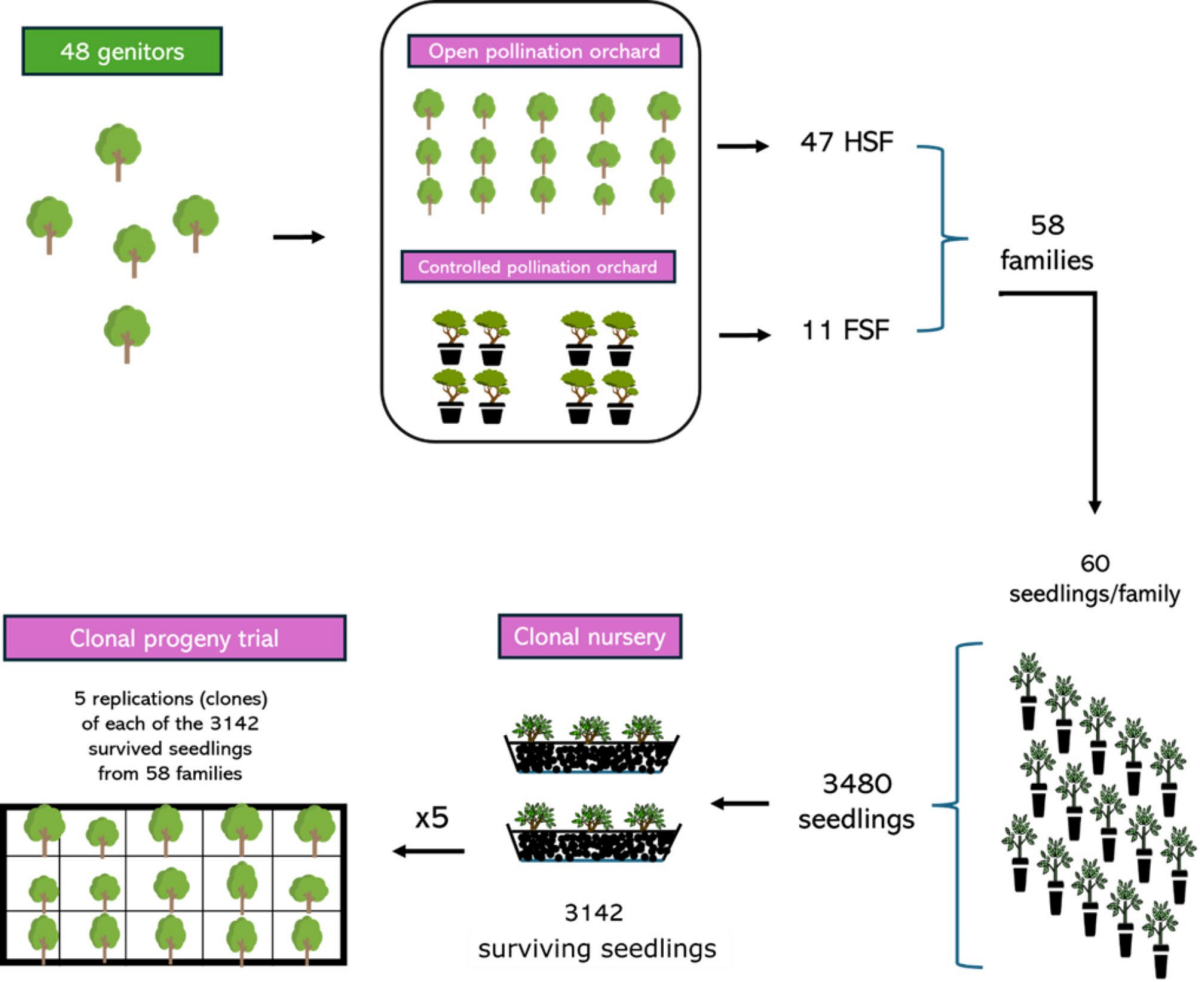
Steps to establish clonal progeny trials (CPT). HSF and FSF stand for half- and full-sibling families, respectively, and x5 indicates that five replications (ramets) of each of the 3142 genotypes were used in each CPT

The genitors chosen to develop the population analyzed in this study included *Eucalyptus grandis*, *Eucalyptus urophylla*, and their hybrids from the Suzano company’s breeding program, located in the state of Mato Grosso do Sul, Brazil. A total of 48 trees (genitors) were selected based on their mean annual increment (MAI; m³ha^− 1^year^− 1^). These genitors were used to generate two types of orchards: (i) an open pollination orchard (OPO); and (ii) a controlled pollination orchard (CPO) (Fig. 1). A total of 58 families were derived from both orchards: 47 from the OPO and 11 from the CPO.

Families derived from the OPO are considered half siblings, while families derived from the CPO are considered full siblings. Seeds from all families were germinated, and 60 seedlings were separated from each family, generating a total of 3,480 genotypes (henceforth called progenies). These were then planted in a clonal nursery for clonal propagation. Of the total number of planted progenies, 3,142 (90.3%) survived and were cloned to establish the clonal progeny trial (CPT) using five replications in two different environments (Fig. 1).

### Experimental design and sites

**Fig. 2:**
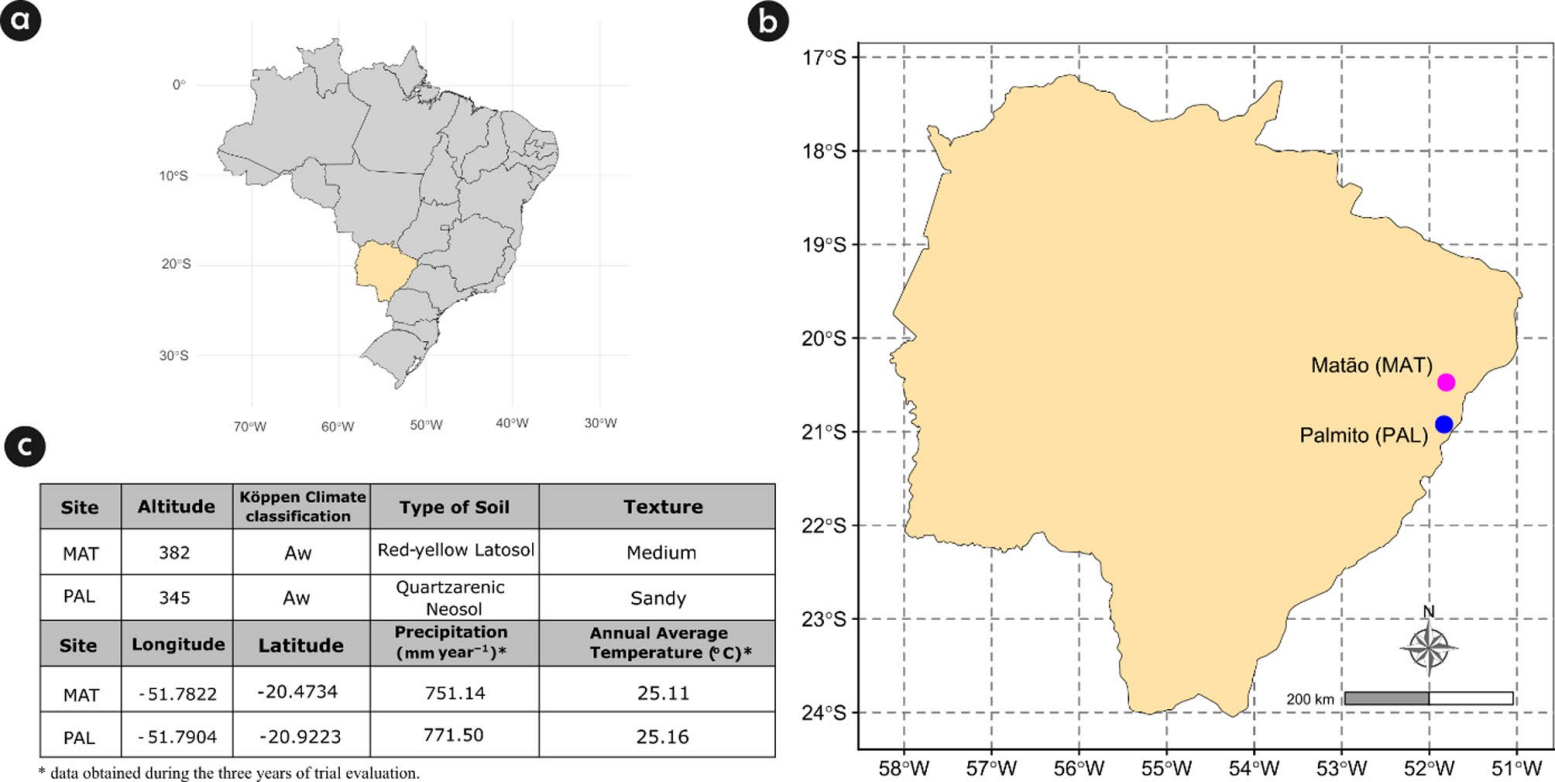
Characteristics of the environment and soil conditions in the two study sites, Matão (MAT) and Palmito (PAL), Mato Grosso do Sul state, Brazil. (**a**) Map of Brazil highlighting the Mato Grosso do Sul state; (**b**) location of the two experimental sites in Mato Grosso do Sul state; and (**c**) environmental and soil characteristics

Two CPTs were planted on two farms (sites) in Mato Grosso do Sul state, Brazil, here referred to Matão (MAT) and Palmito (PAL). The climate at both sites is classified as Aw, which means tropical with dry winters [29]. The biggest differences between trial locations are related to soil type and texture (Fig. 2; Figure S1).

**Fig. 3:**
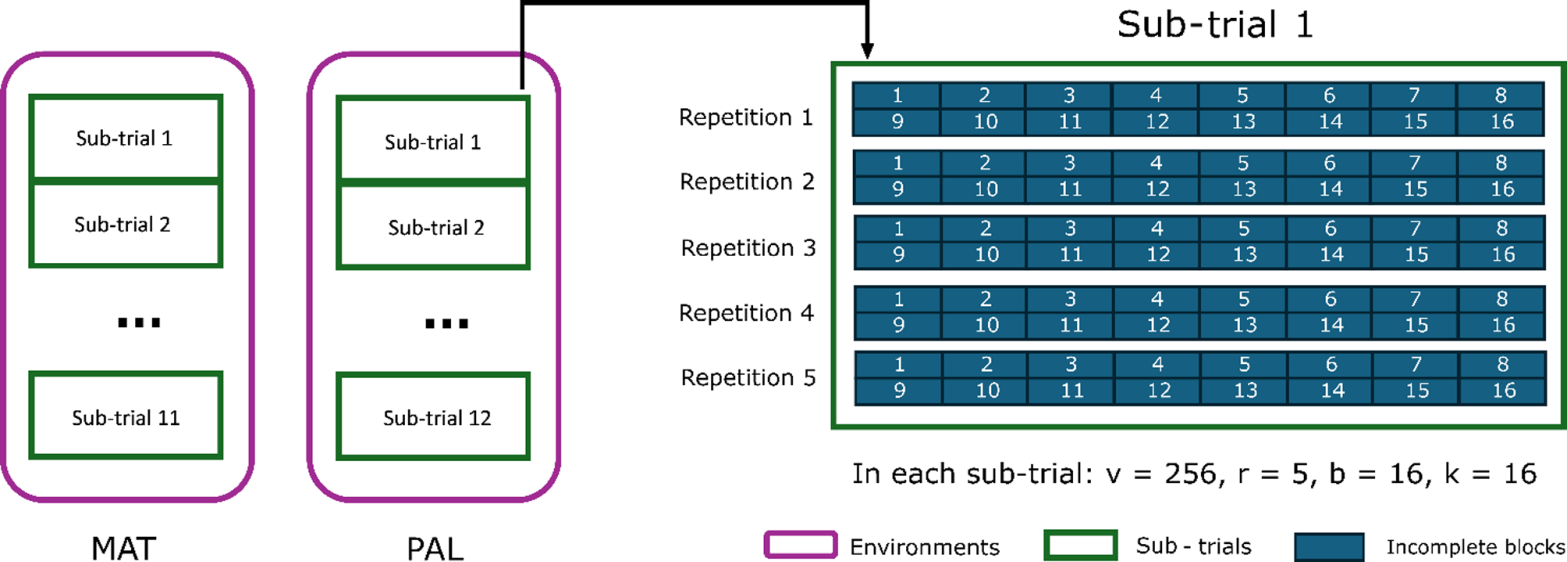
Diagram of the experimental design adopted for the clonal progeny trial (CPT). In each sub-trial, *v* is the number of genotypes, *r* is the number of repetitions, *b* is the number of blocks, *k* is the block size

Each CPT was subdivided into sub-trials (Fig. 3). In environment 1 (MAT), 11 sub-trials were established, while 12 sub-trials were established in environment 2 (PAL). Within each sub-trial, treatments were implemented following an alpha-lattice design (with balanced repetitions and unbalanced blocks). Each sub-trial contained 256 treatments, consisting of two groups of eucalypt genotypes: group 1–250 of the 3,142 progenies; and group 2 - six commercial clones, used as a control. A total of five repetitions were used for each individual genotype within each sub-trial. Therefore, in each sub-trial, five replicates (clones) of each of the 256 treatments were cultivated. Each tree was planted in an individual plot (single tree plot - STP). Within each repetition, the plots (STP) were grouped into 16 incomplete blocks per replication, with each incomplete block containing 16 genotypes (trees) randomly assigned. Therefore, each repetition was segmented into 16 incomplete blocks of 16 genotypes each, an alpha-lattice 16 × 16 configuration necessary to complete the 256 treatments (Fig. 3).

Almost all the 3,142 clones were established in each site, with 2,750 clones evaluated in MAT and 3,000 in PAL. Therefore, a total of 14,080 and 15,360 trees were planted in MAT and PAL sites, respectively. The distance between trees in the rows was 2.5 m and 3.6 m between the rows. To conduct the trials, Suzano’s site management guidelines were followed, including the application per hectare of 1,800 kg of dolomitic limestone for pH correction, 400 kg of 12-20-16 NPK base fertilization, 280 kg of 10-00-27 NPK top dressing fertilization, and weed removal.

### Measurements

All trees were measured at 36 months after planting for circumference at breast height (CBH, cm) and total height (TH, m). A measuring tape (*Vonder*) was used to measure CBH, and total height estimates were measured with an electronic clinometer (*Haglof*). The total individual volume (VOL, m³) with bark was calculated as:

$$\:VOL=\frac{\left(CBH^{2} \pi\:\right)}{40000}\:TH*ST,$$

where CBH is the circumference at breast height (cm); $\:\pi\:$ is the value of pi; TH is the total height (m), and ST is the stem taper (0.45). Volume was converted from m³ to dm³, then standardized as:

$$\:{z}_{i}=\frac{{x}_{i}-{\mu}_{x}\:}{{\sigma}_{x}},$$

where $\:{z}_{i}$ is the standardized volume of the *i*-th observation, $\:{x}_{i}$ is the value of the *i*-th observation, $\:{\mu}_{x}$ and $\:{\sigma}_{x}$ are the mean and standard deviation for volume in dm³, respectively.

### Genotypes

Approximately 80% of the trees were genotyped (2,495 of 3,142 cloned progenies + 6 controls). The genotyped trees include cloned progenies, their parents, and the controls. Two genotyping platforms were used: (1) the Axiom Eucalyptus Genotyping Array (*Axiom Euc72K*) by Thermo Fisher Scientific (Santa Clara, CA, USA), which contains 68,055 SNP markers, used to genotype 1,964 trees; and (2) the Illumina EuCHIP60K [30] by GENESEEK Inc. (Lincoln, NE, USA) with 64,639 SNPs, used to genotype the remaining 531 trees. Only markers common to both genotyping platforms were considered (*N* = 28,177). Additionally, SNP markers exhibiting suboptimal clustering quality were systematically removed from the dataset. For the Thermo Fisher platform, markers that fell under the categories of “Off-Target Variant” (OTV) and “Other” were discarded, resulting in 4,252 SNPs being removed from the dataset. For the Illumina platform, SNPs with a GenTrain score lower than 0.7 were excluded, resulting in 21,371 discarded SNPs.

Finally, low-quality SNPs were filtered to avoid any bias in subsequent analyses. Genotype quality control was performed by applying the following molecular marker exclusion criteria: non-autosomal; duplicate position; call rate < 95% (rate of genotype determination); minor allele frequency (MAF) < 2%; and samples with a call rate < 80%. The PLINK v.1.9 software [31] was used to conduct quality control for each individual panel, and only shared markers were considered. In summary, the remaining dataset included 2,495 genotyped samples and 15,490 SNP markers.

## Relationship matrices

### Corrected pedigree matrix (Ac)

For the genotyped clones, the IBD values between progenies and parents were calculated using the PLINK v.1.9 software [31]. Large-scale genotyping methods are susceptible to genotyping errors due to instrumental factors, the condition of the biological material, and/or sample processing [32]. As such, genotyping errors were considered and a Mendelian Error (ME) rate of up to 1% was accepted. ME is characterized by the observation of marker variants in the progeny that are absent in the parental lines; these variants should not have been biologically segregated and may indicate genotyping errors. Pedigree validation and correction were performed using the “ghap.relfind” function from the GHap R package (Version 3.0.0) [33]. Discrepancies between genomic and pedigree-based relationships were identified and used to correct the pedigree. Undeclared relationships detected through genomic data were incorporated into the updated pedigree to improve its accuracy for subsequent analyses.

### Genomic relationship matrices

The genomic relationship matrix ($\:{\mathbf{G}}_{\mathbf{a}}$) was estimated as follows [34]:

$$\:{G}_{a}=\:\frac{Z{Z}^{{\prime\:}}}{2{\sum}_{j=1}^{n}{p}_{j\:(1-{p}_{j})}},$$

where $\:Z$ represents a matrix of centered marker genotypes for all genotypes, as $\:{Z}_{ij=}\left\{2-2{p}_{j},\:1-2\:{p}_{j},\:-2{p}_{j}\right\}$ [35]. The $\:{Z}_{ij}$ is the element of the *i*-th row and *j*-th column of the marker matrix, and $\:{p}_{j}$ is the frequency of the second most frequent allele at locus *j*. The calculation of $\:Z$ involves transforming the matrix of nucleotide marker genotypes into 0, 1, and 2, with 0 being the most frequent homozygote, 1 the heterozygote, and 2 the second most frequent homozygote.

The dominance genomic relationship matrix ($\:{G}_{d}$) was obtained following [35]:

$$\:{G}_{d}=\:\frac{S{S}^{{\prime\:}}}{4{\sum}_{j=1}^{n}{\left({p}_{j\:\left(1-{p}_{j}\right)}\right)}^{2}},$$

where $\:S$ represents a matrix of dominance deviations for each genotype at each molecular marker, as $\:{S}_{ij=}\left\{-2{\left(1-{p}_{j}\right)}^{2},\:2{p}_{j}\left(1-{p}_{j}\right),\:-2{p}_{j}^{2}\:\:\right\}$ [35]. $\:{S}_{ij}$ is the element of the *i*-th row and *j*-th column of the $\:S$ dominance matrix.

The $\:{\mathbf{G}}_{\mathbf{a}}$ matrix was singular and non-invertible which could be due to the structure of the dataset: the number of markers is greater than the number of observations and there is similarity among genotypes (e.g., clones) [36]. To avoid this issue, it was blended with a submatrix of the numerator relationship matrix from the corrected pedigree [34].

$$\:{{G}_{a}}^{*}=\alpha\:{\mathbf{G}}_{\mathbf{a}}+(1-\alpha\:){\mathbf{A}}_{22}$$

where $\:{{\mathbf{G}}_{\mathbf{a}}}^{*}$ is the blended $\:{\mathbf{G}}_{\mathbf{a}}$ matrix, $\:{\varvec{A}}_{22}$ is the submatrix of $\:{\varvec{A}}_{\varvec{c}}$, including the genotyped clones; *α* is the proportion of genetic variance attributed to the SNP effects, being $\:\alpha\in \left[\text{0,1}\right]$. In this study, $\:\alpha\:$ was defined as *α* = 0.98.

### Statistical analysis

Due to the complexity of the data structure mentioned earlier, a stage-wise analysis was performed.

#### First-stage analysis

In the first stage, the following model was fitted within each trial and environment combination (a total of 23 combinations):

$$\:\varvec{y}={\varvec{X}}_{1}\varvec{g}+\:{\varvec{Z}}_{1}\varvec{r}+\:{\varvec{Z}}_{2}\varvec{b}+\varvec{\xi\:},$$

where $\:\varvec{y}$ represents the vector of phenotypic records; $\:\varvec{g}$ represents the fixed effect vector of genotypes and incidence matrix $\:{\varvec{X}}_{1}$; $\:\varvec{r}$ represents the random effect vector of replication effects with $\:\varvec{r}\:\sim\varvec{N}(0,\:{\varvec{\sigma\:}}_{\varvec{r}}^{2}\:\varvec{I})$ and an incidence matrix $\:{\varvec{Z}}_{1}$, and $\:{\varvec{\sigma\:}}_{\varvec{r}}^{2}$ being the variance of the replications and $\:\varvec{I}$ the incidence matrix; $\:\varvec{b}$ represents the random effect vector of incomplete block effects with $\:\varvec{b}\:\sim\varvec{N}(0,\:{\varvec{\sigma\:}}_{\varvec{b}}^{2}\:\varvec{I})$ and an incidence matrix $\:{\varvec{Z}}_{2}$, and $\:{\varvec{\sigma\:}}_{\varvec{b}}^{2}$ being the variance of the blocks; and $\:\varvec{\xi\:}$ represents correlated residuals in a column-row structure with $\:\varvec{\xi\:}\:\sim\varvec{N}(0,\:{\varvec{\sigma\:}}_{\varvec{\xi\:}}^{2}\:\left[\varvec{A}\varvec{R}1{\varvec{\rho\:}}_{\varvec{c}}\otimes\:\:\varvec{A}\varvec{R}1{\varvec{\rho\:}}_{\varvec{r}}\right]\:\otimes\:\varvec{I})$, and $\:{\varvec{\sigma\:}}_{\varvec{\xi\:}}^{2}$ being the variance or spatially dependent residuals, and $\:\varvec{A}\varvec{R}1{\varvec{\rho\:}}_{\varvec{c}}$ and $\:\varvec{A}\varvec{R}1{\varvec{\rho\:}}_{\varvec{r}}$ the first-order autoregressive correlation matrices for columns and rows, respectively [37]. The operator ⊗ represents the Kronecker product.

#### Second-stage analysis

In the second stage, the following linear mixed model was fitted across environments using the vector of adjusted means from the first stage ($\:{\stackrel{-}{\varvec{y}}}_{1}$):

$$\:{\stackrel{-}{\varvec{y}}}_{1}={\varvec{X}}_{2}\varvec{\beta\:}+\:{\varvec{Z}}_{3}\varvec{t}+\:{\varvec{Z}}_{4}\varvec{a}+\:{\varvec{Z}}_{5}\varvec{d}++\:{\varvec{Z}}_{6}\varvec{j}+\:{\varvec{Z}}_{7}\varvec{k}+\:\varvec{e},$$

where $\:\varvec{\beta\:}$ represents the fixed effect vector of site (MAT and PAL) with design matrix $\:{\varvec{X}}_{2}$; $\:\varvec{t}$ represents the random vector of trials within environment effects with $\:\varvec{t}\:\sim\varvec{N}(0,\:{\varvec{\sigma\:}}_{\varvec{t}}^{2}\:\varvec{I})$ and an incidence matrix $\:{\varvec{Z}}_{3}$, and $\:{\varvec{\sigma\:}}_{\varvec{t}}^{2}$ being the variance of trials within environment effect; $\:\varvec{a}$ is the random effect vector of additive genetic effects with $\:\varvec{a}\:\sim\varvec{N}(0,\:{\varvec{\sigma\:}}_{\varvec{a}}^{2}{\:\varvec{G}}_{\varvec{a}})$ and incidence matrix $\:{\varvec{Z}}_{4}$, and $\:{\varvec{\sigma\:}}_{\varvec{a}}^{2}$ being the variance for the additive genetic effects; $\:\varvec{d}$ is the random effect vector of dominance genetic effects with $\:\varvec{d}\:\sim\varvec{N}(0,\:{\varvec{\sigma\:}}_{\varvec{d}}^{2}{\:\varvec{G}}_{\varvec{d}})$ and incidence matrix $\:{\varvec{Z}}_{5}$, and $\:{\varvec{\sigma\:}}_{\varvec{d}}^{2}$ being the variance for the dominance genetic effects; $\:\varvec{j}$ represents the random vector of interaction between additive genetic and environment effects with $\:\varvec{j}\:\sim\varvec{N}(0,\:{\varvec{\sigma\:}}_{\varvec{j}}^{2}{\:\varvec{G}}_{\varvec{a}}\:\otimes\:\varvec{I}\:)$ and incidence matrix $\:{\varvec{Z}}_{6}$, and $\:{\varvec{\sigma\:}}_{\varvec{j}}^{2}$ being the variance for the interaction between additive genetic and environment effects; $\:\varvec{k}$ represents the random vector of interaction between dominance genetic and environment effects with $\:\varvec{k}\:\sim\varvec{N}\left(0,\:{\varvec{\sigma\:}}_{\varvec{k}}^{2}{\:\varvec{G}}_{\varvec{d}}\:\otimes\:\varvec{I}\:\right)$and incidence matrix $\:{\varvec{Z}}_{7}$, and $\:{\varvec{\sigma\:}}_{\varvec{k}}^{2}$ being the variance for the interaction between dominance genetic and environment effects; and $\:\varvec{e}$ represents the random vector of residuals with $\:\varvec{e}\:\sim\varvec{N}(0,\:{\varvec{\sigma\:}}_{\varvec{e}}^{2}\:\varvec{I})$, and $\:{\sigma}_{e}^{2}$ being the residual variance.

### Genetic parameters

After the second stage, narrow-sense heritability ($\:{h}_{a}^{2}$), coefficient of determination for dominance effects ($\:{\delta\:}^{2}$), and broad-sense heritability ($\:{H}^{2}$) were calculated as: $\:{h}_{a}^{2}={\sigma}_{A}^{2}/{\sigma}_{P}^{2}$; $\:{\delta\:}^{2}={\sigma}_{D}^{2}/{\sigma}_{P}^{2}$; and $\:{H}^{2}=({\sigma}_{a}^{2}+\:{\sigma}_{D}^{2})/{\sigma}_{P}^{2}$, respectively, considering $\:{\sigma}_{P}^{2}={\sigma}_{A}^{2}+\:{\sigma}_{D}^{2}+\:{\sigma}_{t}^{2}+\:{\sigma}_{\text{j}}^{2}+\:{\sigma}_{\text{k}}^{2}+{\sigma}_{\text{e}}^{2}$.

The accuracies of additive genetic effects ($\:{r}_{\widehat{a}a}$) and dominance genetic effects ($\:{r}_{\widehat{d}d}$) were estimated as $\:{r}_{\widehat{a}a}=\sqrt{1-\frac{PEVa}{{\sigma}_{A}^{2}}}$ and $\:{r}_{\widehat{d}d}=\sqrt{1-\frac{PEVd}{{\sigma}_{D}^{2}}}$, respectively,

where $\:PEVa$ and $\:PEVd$ are the prediction error variance derived from the mean of the diagonal elements based on the inverse mixed model equation coefficient matrix for additive and dominance genetic effects. The reliability of additive genetic effects ($\:{r}_{\widehat{a}a}^{2}$) and dominance genetic effects ($\:{r}_{\widehat{d}d}^{2})\:$is similar to those shown above without the square root and considering the prediction error variance of each clone, which pertains to each element in the diagonal of the inverse coefficient matrix.

We selected the 30 best-ranked genotypes for each model. The rate of change in response to selection for the standardized data ($\:{\varDelta}_{R}$) was given as:

$$\:{\varDelta}_{R}=\:S*{H}^{2},$$

where the selection differential ($\:S$) is equal to $\:S\:=i*\:{\sigma}_{p}$, with $\:i$ being the standardized mean of the selected group or the mean of the deviation from the population mean, measured in units of the phenotypic standard deviation of the population ($\:{\sigma}_{p}$).

### Influence of the number of repetitions on heritability and accuracy

The impacts of reducing the number of clonal replicates on heritability and accuracy was assessed considering one to five repetitions for both sites (MAT and PAL). Since we are working with cloned progenies, each evaluated treatment is replicated five times within each sub-trial in each site (Fig. 3). Each of these replicates are present in one repetition of the sub-trial. Therefore, for both sites, we selected all possible combinations of repetitions of size five to one in order to estimate an average heritability and accuracy for each number of replications. For example, by using only two repetitions (size 2) out of five, 10 possible combinations may be obtained for each site. The reported heritability and accuracy if using two repetitions (size 2) out of five, is an average of the 10 combinations. For each combination, the following single-stage linear mixed model was fitted for volume (dm³), with subscript “s” representing components of variance and genetic parameters for the single stage model:

$$\:\varvec{y}=\varvec{X}\varvec{\beta\:}+\:{\varvec{W}}_{1}\varvec{b}+\:{\varvec{W}}_{2}\varvec{a}+\:{\varvec{W}}_{3}\varvec{d}+\varvec{e},$$

where $\:\varvec{y}$ is the vector of phenotypic records; $\:\varvec{\beta\:}$ is the fixed effect vector of site (MAT and PAL), sub-trial within trial, and repetition within sub-trial effects with design matrix $\:\varvec{X}$; $\:\varvec{b}$ is the random effect vector of incomplete block effects with $\:\varvec{b}\:\sim\varvec{N}(0,\:{\varvec{\sigma\:}}_{{\varvec{b}}_{\varvec{s}}}^{2}\:\varvec{I})$ and an incidence matrix $\:{\varvec{W}}_{1}$; $\:\varvec{a}$ is the random effect vector of additive genetic effects with $\:\varvec{a}\:\sim\varvec{N}(0,\:{\varvec{\sigma\:}}_{{\varvec{a}}_{\varvec{s}}}^{2}{\:\varvec{G}}_{\varvec{a}})$ and incidence matrix $\:{\varvec{W}}_{2}$; $\:\varvec{d}$ is the random effect vector of dominance genetic effects with $\:\varvec{d}\:\sim\varvec{N}(0,\:{\varvec{\sigma\:}}_{{\varvec{d}}_{\varvec{s}}}^{2}{\:\varvec{G}}_{\varvec{d}})$ and incidence matrix $\:{\varvec{W}}_{3}$; and $\:\varvec{e}$ is the random effect vector of residual effects with $\:\varvec{e}\:\sim\varvec{N}(0,\:{\oplus}_{\varvec{i}=1}^{2}{\varvec{\sigma\:}}_{{\varvec{e}\varvec{i}}_{\varvec{s}}}^{2})$. $\:{\sigma}_{{a}_{s}}^{2}$ is the additive genetic variance, $\:{\sigma}_{{d}_{s}}^{2}$ is the dominance genetic variance, $\:{\sigma}_{{b}_{s}}^{2}$ is the incomplete block variance, $\:{\sigma}_{{e1}_{s}}^{2}$ is the residual variance for site 1 (MAT), and $\:{\sigma}_{{e2}_{s}}^{2}$ is the residual variance for site 2 (PAL). $\:\varvec{I}$ is an identity matrix,$\:{\:\varvec{G}}_{\varvec{a}}$ is the additive genomic relationship matrix,$\:{\:\varvec{G}}_{\varvec{d}}$ is the dominance genomic relationship matrix, and $\:\oplus\:$ is the direct sum of residual matrices among environments, resulting in a block-diagonal heterogeneous residual covariance matrix.

The narrow-sense heritability for the single stage model ($\:{h}_{{a}_{s}}^{2}$), coefficient of determination for dominance effects for the single stage model ($\:{{\delta}_{s}}^{2}$), and broad-sense heritability for the single stage model ($\:{{H}_{s}}^{2}$) were given as: $\:{h}_{{a}_{s}}^{2}={\sigma}_{{a}_{s}}^{2}/{\sigma}_{{p}_{s}}^{2}$; $\:{{\delta}_{s}}^{2}={\sigma}_{{d}_{s}}^{2}/{\sigma}_{{p}_{s}}^{2}$; and $\:{{H}_{s}}^{2}\:=({\sigma}_{{a}_{s}}^{2}+\:{\sigma}_{{d}_{s}}^{2})/{\sigma}_{{p}_{s}}^{2}$, respectively, considering $\:{\sigma}_{{p}_{s}}^{2}={\sigma}_{{a}_{s}}^{2}+\:{\sigma}_{{d}_{s}}^{2}+\:{\sigma}_{{b}_{s}}^{2}+(\:{\sigma}_{{e1}_{s}}^{2}+\:{\sigma}_{{e2}_{s}}^{2})/2$. The accuracies of additive genetic effects ($\:{r}_{{\widehat{a}a}_{s}}$) and dominance genetic effects ($\:{r}_{{\widehat{d}d}_{s}}$) were estimated as shown previously.

The 10, 20, and 30 best-ranked clones were selected for each combination within each number of replications. The selected clones can differ between combinations, therefore the sum of rankings proposed by Mulamba and Mock [38] was applied to define a final ranking for each number of replications. To track changes in the ranking across the number of replications, the ranking coincidence in percentage, $\:R\:\left(\%\right)$, was calculated as follows:

$$\:R\:\left(\%\right)=\frac{C}{S}*100,$$

where, 𝐶 is the number of coincident genotypes in each number of replicates, and 𝑆 is the number of selected genotypes ranked based on the genotypic value (a + d) (10, 20, or 30).

All analyses were conducted in the R software environment (version 4.3.1) [39]. Linear mixed models were fitted using ASReml-R (version 4.2) [40], while figures were created using the ggplot2 package [41]. The scripts used in this study are available at the Github repository: https://github.com/filipe-manoel/TPC.

## Results

### Genetic parameter estimates by fitting a stage-wise model

In the first stage, the average correlation between columns for the sub-trials in MAT and PAL was 0.01 and − 0.03, respectively. For the rows, the average correlations were − 0.06 for MAT and − 0.11 for PAL. At the second stage, genetic parameters were estimated by modeling additive and dominance genetic effects via a linear mixed model considering these effects. The genetic parameters showed that 15% of the phenotypic variation for volume (dm^3^) can be attributed to additive genetic effects, i.e., heritable genetic effects (Table 1). The dominance genetic effects played a crucial role in explaining the $\:{\sigma}_{p}^{2}$, accounting for 22%. The sub-trial within environment effects explained approximately 48% of the phenotypic variation, while the sum of genotype x environment interactions explained only about 2%. The remaining 12% were attributed to residual effects (random error or noise in the data). Therefore, the narrow-sense ($\:{h}_{a}^{2}$) and broad-sense heritability ($\:{H}^{2}$) for these clonal progeny trials were 0.15 and 0.37, respectively. It is worth noting that the magnitude of the standard error (from 0.03 to 0.06) suggests that the estimates are reliable, which is corroborated by $\:{r}_{\widehat{a}a}$ and $\:{r}_{\widehat{d}d}$ accuracy rates equal to 0.81 and 0.82, respectively.

By selecting the 30 best-ranked genotypes based on the genotypic value (a + d) at 36 months, the response to selection was 97.04%. Thus, there is potential to almost double the volume in future breeding populations by applying early selection at 36 months (Table 1).

**Table 1:** Estimates of the variance components and genetic parameters for the stage-wise analysis

Component	Estimate
$\:{\sigma}_{a}^{2}$	250.76 (31.72)
$\:{\sigma}_{d}^{2}$	365.14 (22.21)
$\:{\sigma}_{t}^{2}$	796.16 (245.95)
$\:{\sigma}_{j}^{2}$	37.47 (7.60)
$\:{\sigma}_{k}^{2}$	6.28 (5.70)
$\:{\sigma}_{e}^{2}$	200.39 (8.91)
$\:{\sigma}_{p}^{2}$	1656.19
$\:{h}_{a}^{2}$	0.15 (0.03)
$\:{\delta\:}^{2}$	0.22 (0.04)
$\:{H}^{2}$	0.37 (0.06)
$\:{r}_{\widehat{a}a}$	0.81
$\:{r}_{\widehat{d}d}$	0.82
$\:{r}_{\widehat{a}a}^{2}$	0.66
$\:{r}_{\widehat{d}d}^{2}$	0.67
$\:R\%$	97.04
$\:\stackrel{-}{{x}_{0}}$	52.36
$\:\stackrel{-}{{x}_{s}}$	113.56

Where: $\:{\sigma}_{a}^{2}$ is the additive genetic variance; $\:{\sigma}_{d}^{2}$ is the dominance genetic variance; $\:{\sigma}_{t}^{2}$ is the trial within environment variance; $\:{\varvec{\sigma\:}}_{\varvec{j}}^{2}$ is the variance for the interaction between additive genetic and environment effects; $\:{\varvec{\sigma\:}}_{\varvec{k}}^{2}$ is the variance for the interaction between dominance genetic and environment effects;

$\:{\sigma}_{e}^{2}$ is the residual variance; $\:{\sigma}_{p}^{2}$ is the phenotypic variance; $\:{h}_{a}^{2}$ is the mean narrow-sense heritability; $\:{\delta\:}^{2}$ is the coefficient of determination for dominance effects; $\:{H}^{2}$ is the broad-sense heritability; $\:{r}_{\widehat{a}a}$ is the accuracy for additive genetic effects; $\:{r}_{\widehat{d}d}$ is the accuracy for dominance genetic effects; $\:{r}_{\widehat{a}a}^{2}$ is the reliability for additive genetic effects; $\:{r}_{\widehat{d}d}^{2}$ is the reliability for dominance genetic effects;

$\:R\%$ is the response to selection in percentage, when selecting the 30 best-ranked genotypes; $\:\stackrel{-}{{x}_{0}}$ is the trial mean, and $\:\stackrel{-}{{x}_{s}}$ is the mean of the selected population; standard deviations are in parenthesis.

### Influence of the number of replications on heritability and accuracy

**Fig. 4:**
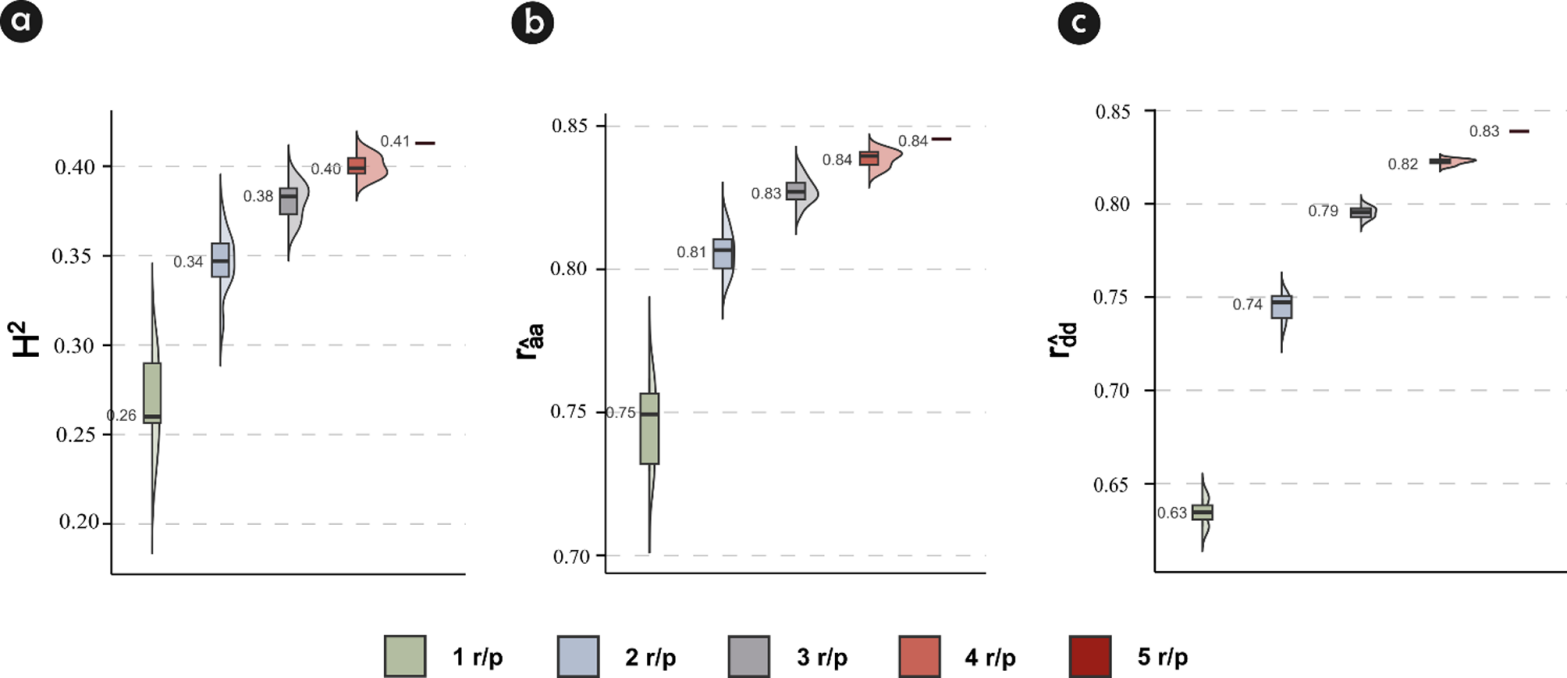
(**a**) Broad-sense heritability (*H²*); (**b**) accuracy for additive genetic effects ($\:{r}_{\widehat{a}a}$); and (**c**) accuracy for dominance effects ($\:{r}_{\widehat{d}d})$ by fitting a single stage genomic model in a two environment trial (MAT and PAL) considering from one to five ramets/progeny (r/p)

For all evaluated scenarios, the estimated broad-sense heritability ($\:{H}^{2}$) for volume (dm^3^) for the adopted joint modeling approach was considered of moderate magnitude (Fig. 4a). When all five replications were used to estimate heritability, we obtained the greatest mean value for *H*^*2*^ and the least dispersion around the mean (Fig. 4a). An opposite trend was observed as the number of replications decreased, with one replication showing results 1.57-fold lower than the results with five replications in both locations. Nevertheless, using three replications instead of five only reduced the mean $\:{H}^{2}$ from 0.41 to 0.38 (Fig. 4a). The accuracy for additive genetic effects ($\:{r}_{\widehat{a}a}$) and for dominance genetic effects ($\:{r}_{\widehat{d}d})$ showed similar patterns when reducing the number of replications. Using four replications instead of five had little impact on the magnitude of $\:{r}_{\widehat{a}a}$ and $\:{r}_{\widehat{d}d}$. Meanwhile, three instead of five replications caused a slight reduction in $\:{r}_{\widehat{a}a}$ from 0.84 to 0.83 and in $\:{r}_{\widehat{d}d}$ from 0.83 to 0.79 (Fig. 4b and c).

**Fig. 5:**
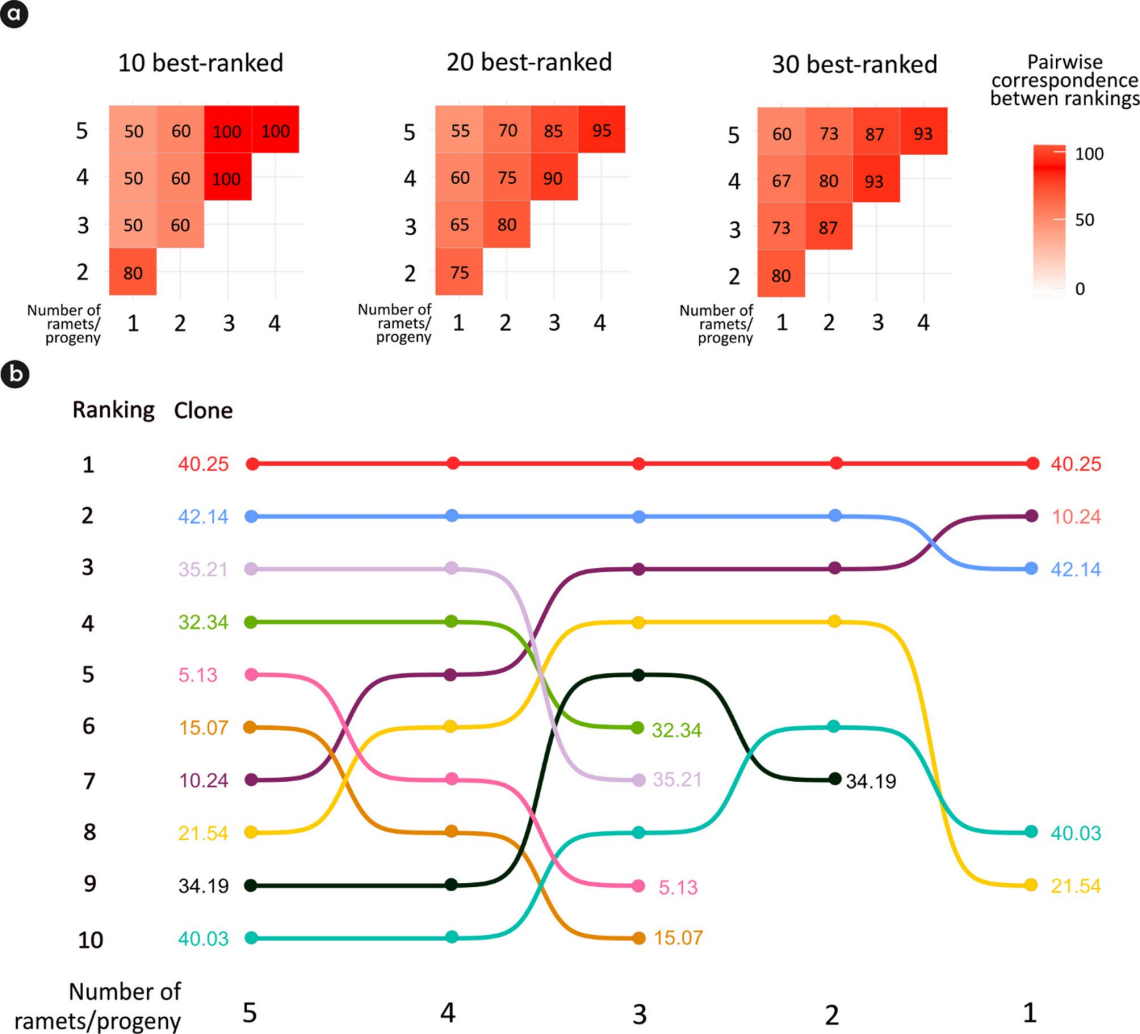
(**a**) Pairwise ranking coincidence by selecting the 10 best-ranked clones (left), 20 best-ranked clones (center), and 30 best-ranked clones (right) by fitting a single-stage genomic model in a two environment trial (MAT and PAL) based on the genotypic value (g = a + d), with the number of ramets/progeny decreasing from five to one; (**b**) changes in the ranking by fitting a single-stage genomic model in a two environment trial (MAT and PAL) as the number of ramets/progeny decreased from five to one. The selected individuals can differ between combinations, therefore the sum of rankings proposed by [38] was applied to define a final ranking for each number of replications

Independently of the number of selected clones (10, 20, or 30) and the number of repetitions, at least half of the clones were always selected (Fig. 5a). Considering a selection of the 10 best-ranked genotypes, the same 10 clones were selected using three, four, and five ramets/progeny (Fig. 5a and b). However, when two ramets/progeny were used instead of five, only six common clones were selected, and when one ramet/progeny was used, only five common clones were selected (Fig. 5b).

To ensure that the 10 best-ranked clones using five ramets/progeny were included in the selection for between four and one ramets/progeny, we should select 10, 10, 24, and 120 clones, respectively (Table 2). These values correspond to the lowest ranking position of the 10 best-ranked genotypes considering five ramets/progeny (Table 2).

**Table 2:** The 10 best-ranked clones using five ramets/progeny (r/p) and their ranking position when considering different numbers of replications

Clone	Ranking Position				
	5 *r*/*p*	4 *r*/*p*	3 *r*/*p*	2 *r*/*p*	1 *r*/*p*
40.25	1	1	1	1	1
42.14	2	2	2	2	3
35.21	3	3	7	24	120
32.34	4	4	6	22	96
5.13	5	7	9	18	49
15.07	6	8	10	21	72
10.24	7	5	3	3	2
21.54	8	6	4	4	9
34.19	9	9	5	7	12
40.03	10	10	8	6	8

## Discussion

Our results emphasize the potential of clonal progeny trials (CPT) to offer efficient and advantageous selection strategies for vegetatively propagated species. This section discusses the findings and contributions made by the present study with regards to: (i) the viability and theoretical considerations for eucalypt clonal selection programs based on the use of CPT; (ii) validating CPT as an effective field design to estimate genetic parameters and predict genotypic effects with high statistical power, exploring its potential to identify top performing progenies that provide promising responses to selection at early breeding stages; and (iii) assessing the feasibility of reducing the number of ramets/progeny in future CPT by quantifying impact on ranking coincidence and genetic parameters.

### Clonal selection programs based on clonal progeny trials

The success of eucalypt breeding in Brazil is largely attributed to reciprocal recurrent selection (RRS) programs [5]. In RRS, pure species eucalypts are crossed to generate hybrid families of half or full siblings, which are then established in hybrid progeny trials. This process is repeated after a cycle of intrapopulation recurrent selection, improving the performance of the hybrids due to the improvement of pure species. Despite the effectiveness of RRS programs, significant investment in time is needed to obtain new commercial genotypes, since information from the hybrid progeny trials is required to adventitiously recombine the pure species. In many eucalypt breeding programs in Brazil, this time limitation can be mitigated by vegetative propagation of the genotypes. Vegetative propagation allows the genetic fixation of both additive and non-additive (e.g., dominance) effects controlling most yield traits at any stage of the breeding program; however, this advantage tends to create a situation in which the breeder is constantly seeking an ideotype to clone. This may pose some risks since hybrid progeny trials were designed to select among progenies and not genotypes within progeny. Among many other factors, such as climate change, pests, and disease, this “ideotype seeking” may be contributing to the current stagnation of eucalypt forest productivity in Brazil.

**Fig. 6:**
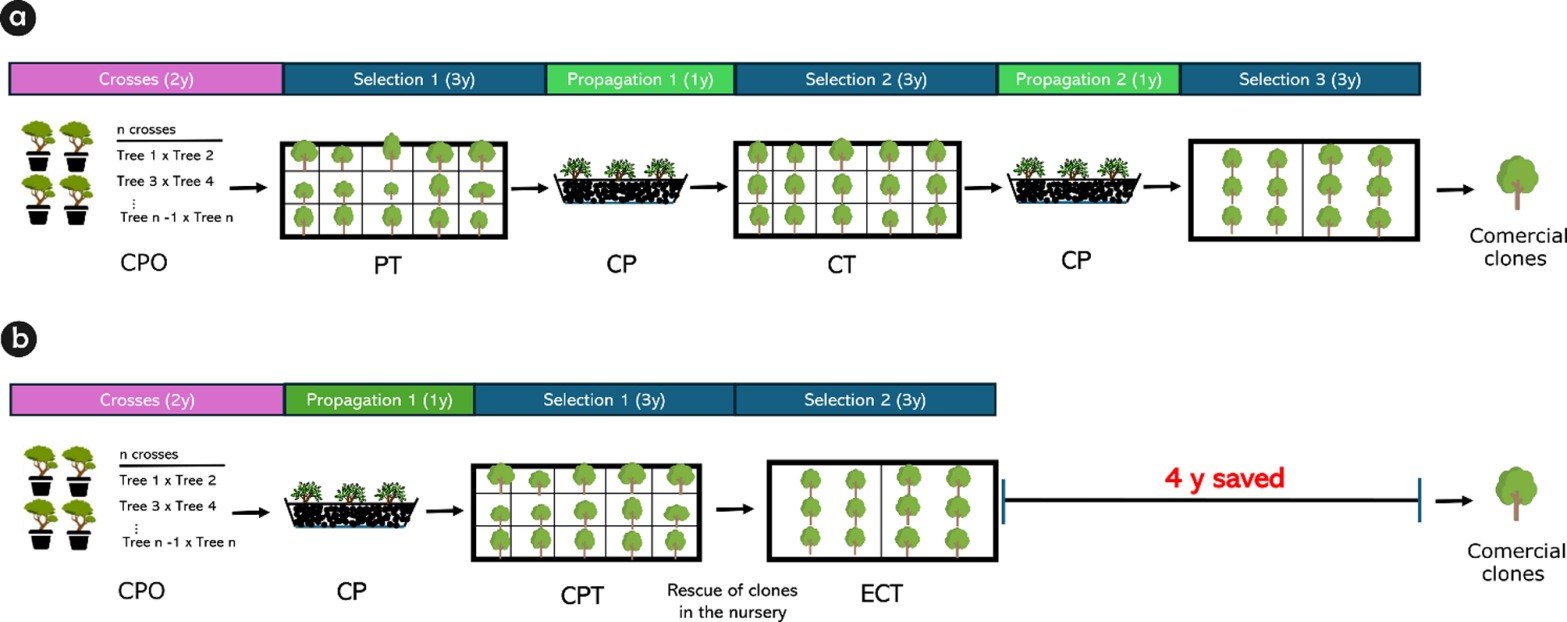
(**a**) Conventional *Eucalyptus* clone selection pipeline; and (**b**) clone selection pipeline based on cloned progeny trial (CPT). CPO = controlled pollination orchard, PT = progeny trial, CP = clonal propagation, CT = clonal trial, ECT = expanded clonal trial, y = year(s)

CPT offers the advantage of evaluating cloned genotypes that were replicated and randomized throughout the trial, which means that the selection unit is the cloned progeny and not the seminal hybrid progeny composed of genetically distinct genotypes. Another advantage of using CPT is that the cloned progenies can be evaluated in many locations and environments used in a breeding program. Depending on the magnitude of genotype x environment (GxE) interaction, this may be a critical success factor in the selection of clones that are better adapted to specific environmental conditions [18]. In a conventional eucalypt clone selection pipeline (Fig. 6a), identifying the GxE interaction at the clone level would only be possible in more advanced stages (clonal stages) of a breeding program, which may pose a challenge to identifying the best performing clones for a specific environment. Also, our findings support the assumption that individual selection at early stages (hybrid progeny trials) is responsible for the poor performance correlation between the genotypes selected during this phase (ortets) and their performance as clones (ramets) [14]. Therefore, CPT enables a more precise assessment of genotypic value of the genotypes that will be tested in the expanded clonal trials (ECT) (Fig. 6b).

The efficiency of clone selection based on CPT can be improved using molecular information. By applying genomic relationship matrices, complex relationships between and within genotypes that are generally overlooked in common breeding routines can be examined to choose crosses in a controlled pollination orchard and then define the most suitable genotypes to be tested in CPT after the clonal propagation stage. Additionally, molecular data helps to reconstruct the pedigree of the evaluated population allowing for the identification of genealogical relationships among individuals [23]. With CPT, molecular information can help reduce bias in estimating genetic parameters, providing more realistic heritability estimates, leading to more accurate predictions of genotypic values and genetic gains [11]. As biotechnological research and innovation develop, it may be possible to discard progenies that are susceptible to oligogenic diseases or other elements that affect productivity and have a molecular signature, which will improve the efficiency of CPT.

In a conventional eucalypt clone selection pipeline, an efficient method involves selecting the best genotypes from pure species populations ideally by recurrent selection and transplanting them in hybridization orchards for controlled crosses and intensive flower production (Fig. 6). Traditional breeding programs require a rigorous process: selected genotypes undergo a progeny test to confirm high performance as clones, followed by production of mini-stumps for cuttings (ramets), and the establishment of large clonal trials (CT) (Fig. 6a). This process can take about 13 years and involves ECT with a large number of ramets to be evaluated at a large scale; it requires substantial investment and time to produce mini-stumps and seedlings. In contrast, a clonal program based on clonal progeny trials (CPT) streamlines this process (Fig. 6b). By incorporating CPT in the clone selection pipeline, the entire process can be shortened from 13 years to nine years, saving approximately four years compared to the conventional pipeline. This reduction is accomplished by enhancing heritability and accuracy at the early stages of breeding, thus speeding up genetic gains. Consequently, a clone selection pipeline using CPT associated with molecular information can make more clones available for commercial plantations more rapidly, enhancing overall efficiency and effectiveness. Furthermore, molecular markers enable the genetic identification of individuals in trials, aiding in the traceability of errors caused by contamination during crossings and potential mechanical mistakes during field test implementation.

### Genetic parameter estimates and response to selection

Genomic relationships consistently provide a more accurate representation of genetic variability compared to relationships based solely on pedigree information. Studies have demonstrated that incorporating genomic data in genetic evaluations and breeding programs leads to more precise estimates of genetic parameters and enhanced selection efficiencies [42–44]. This approach offers a more reliable assessment of genetic relatedness among genotypes. By leveraging genomic relationships, breeders can identify superior genotypes more accurately, leading to increased genetic gains and more efficient breeding programs [42, 44]. When applying the stage-wise model, a preponderance of dominance over additive variance for volume was identified, which is reflected in the magnitude of heritability. This effect is already widely known and occurs because the expression of dominance effects depends greatly on heterozygosity [45, 46], which is common in hybrid populations such as the one studied herein (*Eucalyptus urophylla* x *Eucalyptus grandis*) [47, 48]).

A single-locus dominance makes minor contributions to traits in hybrid populations indicates that the cumulative effect of multiple loci exhibiting dominance can lead to substantial variance in dominance within hybrids [49]. In addition, the additive plus dominance genomic models are a viable approach to enhance genetic performance in crossbred populations with substantial dominance genetic variation, underscoring the importance of considering dominance effects in improving breeding outcomes [50]. This supports our choice of model since incorporating dominance effects in the genetic evaluations of this population led to higher accuracy estimates for volume. Dominance effects can help to explain the variation in growth traits such as volume for eucalypts, as previously reported [6, 47, 48, 51]. Early selection at three years of age has proven to be efficient for eucalypt breeding programs in Brazil [52]. This occurs due to the high genetic correlation (greater than 0.90) between early ages (three years of age) and harvesting (five or six years of age) [53, 54], which is even more pronounced when selection is conducted with high accuracy (greater than 0.80) [55].

### Influence of the number of ramets/progeny on heritability and accuracy

Cloning genotypes within progeny tends to increase precision in estimates of variance components, resulting in more reliable heritability estimates [18, 56, 57]. Our results suggest that a reduction in the number of ramets/progeny will have a minimal impact on estimates but a large operational gain in resources and time (Fig. 4). Santos et al. [18] suggested that five repetitions of a genotype may be sufficient for this stage of clone selection, since the selected genotypes will be tested as clones with a greater number of trees per plot and environments. However, the authors evaluated 5, 10, 15, 20, 25, and 30 repetitions per clone, meaning that five repetitions must be clearly very favorable for breeders, as more repetitions per genotype lead to better estimates of genetic parameters. Nevertheless, a large number of repetitions is impractical due to the extensive work involved in implementing a CPT in the field with a large number of candidate genotypes.

In general, the heritability and accuracy values were lower when one repetition of the genotype was considered compared to three repetitions. In traditional progeny trials genotypes have only one repetition as they come from seeds, resulting in the poor correlation often found between performance of genotypes selected in traditional progeny trials (PT) and expanded clonal trials (ECT). To ensure that the 10 best-ranked clones based on three repetitions of the genotype are also selected when using only one repetition, the top 120 clones should be selected, which means increasing the selection intensity by 1200%. The evidence suggests that poor performance correlation is due to the lack of repetition in PT, which leads to poor precision in partitioning genetic variance and estimating the genotypic values ​​of the candidate genotypes within progeny at this stage [11, 15]. Therefore, we believe that at least three repetitions of the genotypes within progeny (ramets/progeny) should be used to mitigate this problem in future breeding programs.

The implementation of CPT will have a positive impact on accuracy. Improving accuracy can provide more reliable selection, allowing for higher selection intensities. Therefore, greater trait heritability helps breeders use higher selection intensities to obtain even greater selection gains. From a practical perspective, CPT has the potential to mitigate the issue of poor correlation between the performance of genotypes in progeny trials and clonal trials [11, 12, 18]. Due to this issue, breeders cannot apply high selection intensities at the first stage of the clone selection program, as good genotypes may be discarded, resulting in a reduction in both genetic gains and success in obtaining new operational clones [14]. We believe that it is possible to overcome this limitation and suggest that the use of three ramets/progeny offers a key advantage that supports the use of CPT in clone selection programs.

CPT offers an innovative and efficient operational process to more accurately evaluate genotypes in the early stages of clone selection programs. CPT can be employed by both private and public institutions to overcome the limitations of traditional progeny trials (poor correlation, accuracy, and no repetition of the genotype per se) and significantly reduce the breeding cycle. Additionally, molecular data can be employed at various stages of CPT clone selection programs to increase its potential for success. The proposed approach not only validates existing research but also sets the stage for more precise and efficient tree clonal selection strategies, ultimately advancing forest genetics.

## Electronic Supplementary Material

Below is the link to the electronic supplementary material.

Supplementary Material 1

## Data Availability

All the data reported in the study is analyzed and presented in tables and figures. Additional datasets used are available from the corresponding author in accordance with the data sharing policies of Suzano S.A.

## References

[1] Soares AAV, Leite HG, Souza AL, Silva SR, Lourenço HM, Forrester DI. Increasing stand structural heterogeneity reduces productivity in Brazilian *Eucalyptus* monoclonal stands. For Ecol Manag. 2016;373:26–32.

[2] Yadav S, Ross EM, Wei X, Powell O, Hivert V, Hickey LT et al. Optimising clonal performance in sugarcane: leveraging non-additive effects via mate-allocation strategies. Front Plant Sci [Internet]. 2023 [cited 2025 Jan 16];14. Available from: https://www.frontiersin.org/journals/plant-science/articles/10.3389/fpls.2023.1260517/full 10.3389/fpls.2023.1260517 PMC10667552 38023905

[3] Wu HX. Benefits and risks of using clones in forestry– a review. Scand J for Res. 2019;34:352–9.

[4] Lima JL, de Souza JC, Ramalho MAP, Andrade HB, de Sousa LC. Early selection of parents and trees in *Eucalyptus* full-sib progeny tests. Crop Breed Appl Biotechnol. 2011;11:10–6.

[5] Simiqueli GF, Resende RT, Takahashi EK, de Sousa JE, Grattapaglia D. Realized genomic selection across generations in a reciprocal recurrent selection breeding program of *Eucalyptus* hybrids. Front Plant Sci. 2023;14:1–11. 10.3389/fpls.2023.1252504 PMC10641691 37965018

[6] Paludeto JGZ, Grattapaglia D, Estopa RA, Tambarussi EV. Genomic relationship–based genetic parameters and prospects of genomic selection for growth and wood quality traits in *Eucalyptus Benthamii*. Tree Genet Genomes. 2021;17:38.

[7] Borralho NMG, Kanowski PJ. Correspondence of performance between genetically related clones and seedlings. Can J Res. 1995;25:500–6.

[8] Bombonato AL, Gouvêa LRL, Verardi CK, Silva GAP, de Souza Gonçalves P. Rubber tree ortet-ramet genetic correlation and early selection efficiency to reduce rubber tree breeding cycle. Ind Crops Prod. 2015;77:855–60.

[9] Wilcox JR, Farmer RE. Heritability and C effects in early root growth of eastern cottonwood cuttings. Heredity. 1968;23:239–45.

[10] Münzbergová Z, Kr˘ivánek M, Bucharová A, Juklíc˘ková V, Herben T. Ramet performance in two tussock plants — do the tussock-level parameters matter? Flora - morphology, distribution. Funct Ecol Plants. 2005;200:275–84.

[11] Shalizi MN, Gezan SA, McKeand SE, Sherrill JR, Cumbie WP, Whetten RW, et al. Correspondence between breeding values of the same *Pinus taeda* L. genotypes from clonal trials and half-sib seedling progeny trials. For Sci. 2020;66:600–11.

[12] Foster GS, Shaw DV. Using clonal replicates to explore genetic variation in a perennial plant species. Theoret Appl Genet. 1988;76:788–94. 24232359 10.1007/BF00303527

[13] Mullin TJ, Morgenstern EK, Park YS, Fowler DP. Genetic parameters from a clonally replicated test of black spruce (Piceamariana). Can J Res. 1992;22:24–36.

[14] Reis CAF, Gonçalves FMA, Rosse LN, Costa RRGF, Ramalho MAP. Correspondence between performance of *Eucalyptus* spp trees selected from family and clonal tests. Genet Mol Res. 2011;10:1172–9. 21732281 10.4238/vol10-2gmr1078

[15] Burdon RD, Shelbourne CJA. Part 6 the use of vegetative propagation for genetic and physiological information. N Z J Forest Sci. 1974;4:1–12.

[16] Costa e Silva J, Borralho NMG, Potts BM. Additive and non-additive genetic parameters from clonally replicated and seedling progenies of *Eucalyptus globulus*. Theor Appl Genet. 2004;108:1113–9. 15067398 10.1007/s00122-003-1524-5

[17] Weng YH, Park YS, Krasowski MJ, Tosh KJ, Adams G. Partitioning of genetic variance and selection efficiency for alternative vegetative deployment strategies for white spruce in Eastern Canada. Tree Genet Genomes. 2008;4:809–19.

[18] Santos HG, de Lima JL, Marçal T, de Siqueira S, de Aguiar L, Ramalho AM. Would it be possible to reduce the number of repetitions in the evaluation of clones in a single tree plot? Euphytica. 2024;220:54.

[19] Burton GW, De Vane de E. Estimating heritability in tall fescue (*Festuca arundinacea*) from replicated clonal material. Agron J. 1953;45:478–81.

[20] Park YS, Fowler DP. Genetic variances among clonally propagated populations of tamarack and the implications for clonal forestry. Can J Res. 1987;17:1175–80.

[21] Fuessley BC, Marsh CM. Method and apparatus for tracking individual plants while growing and/or after harvest [Internet]; 2010 [cited 2025 Jan 16]. Available from: https://patents.google.com/patent/US7702462B2/en

[22] Munoz PR, Resende MFR, Huber DA, Quesada T, Resende MDV, Neale DB, et al. Genomic relationship matrix for correcting pedigree errors in breeding populations: impact on genetic parameters and genomic selection accuracy. Crop Sci. 2014;54:1115–23.

[23] Klápště J, Suontama M, Dungey HS, Telfer EJ, Graham NJ, Low CB, et al. Effect of hidden relatedness on single-step genetic evaluation in an Advanced Open-pollinated breeding program. J Hered. 2018;109:802–10. 30285150 10.1093/jhered/esy051 PMC6208454

[24] Tambarussi EV, Pereira FB, da Silva PHM, Lee D, Bush D. Are tree breeders properly predicting genetic gain? A case study involving *Corymbia* species. Euphytica. 2018;214:150.

[25] Costa e Silva J, Borralho NMG, Araújo JA, Vaillancourt RE, Potts BM. Genetic parameters for growth, wood density and pulp yield in *Eucalyptus globulus*. Tree Genet Genomes. 2009;5:291–305.

[26] Cappa EP, Ratcliffe B, Chen C, Thomas BR, Liu Y, Klutsch J, et al. Improving lodgepole pine genomic evaluation using spatial correlation structure and SNP selection with single-step GBLUP. Heredity. 2022;128:209–24. 35181761 10.1038/s41437-022-00508-2 PMC8986842

[27] Allen JP, Snitkin E, Pincus NB, Hauser AR. Forest and trees: exploring bacterial virulence with genome-wide Association studies and Machine Learning. Trends Microbiol. 2021;29:621–33. 33455849 10.1016/j.tim.2020.12.002 PMC8187264

[28] Zapata-Valenzuela J, Isik F, Maltecca C, Wegrzyn J, Neale D, McKeand S, et al. SNP markers trace familial linkages in a cloned population of *Pinus taeda*—prospects for genomic selection. Tree Genet Genomes. 2012;8:1307–18.

[29] Alvares CA, Stape JL, Sentelhas PC, de Moraes Gonçalves JL, Sparovek G. Köppen’s climate classification map for Brazil. Meteorol Z. 2013:711–28.

[30] Silva-Junior OB, Faria DA, Grattapaglia D. A flexible multi‐species genome‐wide 60K SNP chip developed from pooled resequencing of 240 *Eucalyptus* tree genomes across 12 species. New Phytol. 2015;206:1527–40. 25684350 10.1111/nph.13322

[31] Chang CC, Chow CC, Tellier LC, Vattikuti S, Purcell SM, Lee JJ. Second-generation PLINK: rising to the challenge of larger and richer datasets. Gigascience. 2015;4:s13742-015-0047–8. 10.1186/s13742-015-0047-8 PMC4342193 25722852

[32] Saunders IW, Brohede J, Hannan GN. Estimating genotyping error rates from mendelian errors in SNP array genotypes and their impact on inference. Genomics. 2007;90:291–6. 17587543 10.1016/j.ygeno.2007.05.011

[33] Utsunomiya YT, Milanesi M, Utsunomiya ATH, Ajmone-Marsan P, Garcia JF. GHap: an R package for genome-wide haplotyping. Bioinformatics. 2016;32:2861–2. 27283951 10.1093/bioinformatics/btw356

[34] VanRaden PM. Efficient methods to compute genomic predictions. J Dairy Sci. 2008;91:4414–23. 18946147 10.3168/jds.2007-0980

[35] Vitezica ZG, Varona L, Legarra A. On the Additive and Dominant Variance and Covariance of individuals within the genomic selection scope. Genetics. 2013;195:1223–30. 24121775 10.1534/genetics.113.155176 PMC3832268

[36] Lourenco D, Legarra A, Tsuruta S, Masuda Y, Aguilar I, Misztal I. Single-step genomic evaluations from theory to practice: using SNP chips and sequence data in BLUPF90. Genes. 2020;11:790. 32674271 10.3390/genes11070790 PMC7397237

[37] Gilmour AR, Cullis BR, Verbyla A. Accounting for natural and extraneous variation in the analysis of field experiments. J Agricultural Biol Environ Stat. 1997;2:269–93.

[38] Mulamba NN, Mock JJ. Improvement of yield potential of the Eto Blanco maize (*Zea mays* L.) population by breeding for plant traits; 1978 [cited 2025 Jan 16]; Available from: https://www.cabidigitallibrary.org/doi/full/10.5555/19791677302

[39] R Core Team. A language and environment for statistical computing. https://www.R-project.org/; 2022.

[40] Butler D, Cullis B, Gilmour AR, Gogel BG, Thompson R. ASReml-R reference manual version 4.2. Hemel Hempstead, HP2 4TP, UK. VSN International Ltd.; 2023.

[41] Wickham H. ggplot2: elegant graphics for data analysis. 2nd ed. Springer; 2016.

[42] Beaulieu J, Lenz P, Bousquet J. Metadata analysis indicates biased estimation of genetic parameters and gains using conventional pedigree information instead of genomic-based approaches in tree breeding. Sci Rep. 2022;12:3933. 35273188 10.1038/s41598-022-06681-y PMC8913692

[43] Sonesson AK, Woolliams JA, Meuwissen TH. Genomic selection requires genomic control of inbreeding. Genet Selection Evol. 2012;44:27. 10.1186/1297-9686-44-27 PMC3522025 22898324

[44] VanRaden PM, Olson KM, Wiggans GR, Cole JB, Tooker ME. Genomic inbreeding and relationships among holsteins, jerseys, and Brown Swiss. J Dairy Sci. 2011;94:5673–82. 22032391 10.3168/jds.2011-4500

[45] Legarra A, Gonzalez-Dieguez DO, Charcosset A, Vitezica ZG. Impact of interpopulation distance on dominance variance and average heterosis in hybrid populations within species. Genetics. 2023;224:iyad059. 37021800 10.1093/genetics/iyad059

[46] Lynch M, Walsh B. Genetics and analysis of quantitative traits. 1st edn. Sunderland, Mass: Sinauer Associates is an imprint of Oxford University Press; 1998.

[47] Garel MEC, Philippe V. Estimating of Additive, Dominance, and Epistatic Genetic Variance in Eucalypt Hybrid Population. Silvae Genetica. 2022;71:39–46.

[48] Lima BM, Cappa EP, Silva-Junior OB, Garcia C, Mansfield SD, Grattapaglia D. Quantitative genetic parameters for growth and wood properties in *Eucalyptus urograndis* hybrid using near-infrared phenotyping and genome-wide SNP-based relationships. Albrectsen BR, editor. PLoS ONE. 2019;14:e0218747. 10.1371/journal.pone.0218747 PMC6590816 31233563

[49] Zhou G, Chen Y, Yao W, Zhang C, Xie W, Hua J et al. Genetic composition of yield heterosis in an elite rice hybrid. Proc Nat Acad Sci. 2012;109:15847–52. 10.1073/pnas.1214141109 PMC3465387 23019369

[50] Nishio M, Satoh M. Including Dominance effects in the genomic BLUP method for genomic evaluation. PLoS ONE. 2014;9:e85792. 24416447 10.1371/journal.pone.0085792 PMC3885721

[51] Mora F, Ballesta P, Serra N. Bayesian analysis of growth, stem straightness and branching quality in full-sib families of *Eucalyptus globulus*. Bragantia. 2019;78:328–36.

[52] Ferreira FM, Chaves SF, da Santos S, Nunes OP, Tambarussi ACP, da Pereira EV. Competition effects can mislead selection in eucalypt breeding trials. For Ecol Manag. 2024;561:121892.

[53] González M, Resquín F, Balmelli G. Estimation of genetic parameters for productive traits in *Eucalyptus tereticornis* and implications for breeding. Bosque (Valdivia). 2023;44:493–502.

[54] Rocha LF, Benatti TR, de Siqueira L, de Souza ICG, Bianchin I, de Souza AJ, et al. Quantitative trait loci related to growth and wood quality traits in *Eucalyptus grandis* W. Hill identified through single- and multi-trait genome-wide association studies. Tree Genet Genomes. 2022;18:38.

[55] Resende MDV, Alves RS. Linear, generalized, hierarchical, bayesian and random regression mixed models in genetic/genomics in plant breeding. Funct Plant Breed J. 2020;2:1–31.

[56] Ramalho MAP, Souza T, da Junior S. VP da S. Intrapopulation recurrent selection strategies in plant breeding. Functional Plant Breeding J. 2023;5:1–11.

[57] Walker TD, Cumbie WP, Isik F. Single-step genomic analysis increases the Accuracy of within-family selection in a clonally replicated Population of *Pinus taeda* L. For Sci. 2022;68:37–52.

