# Supplementary material for: Accelerating eucalypt clone selection pipeline via cloned progeny trials and molecular data
Source: Plant Methods. 2025 Feb 14;21:19. doi: 10.1186/s13007-025-01342-3 (PMC11827336; doi:10.1186/s13007-025-01342-3)
Supplement: Supplementary file 1 — Supplementary Material 1 [file 13007_2025_1342_MOESM1_ESM.docx]

**
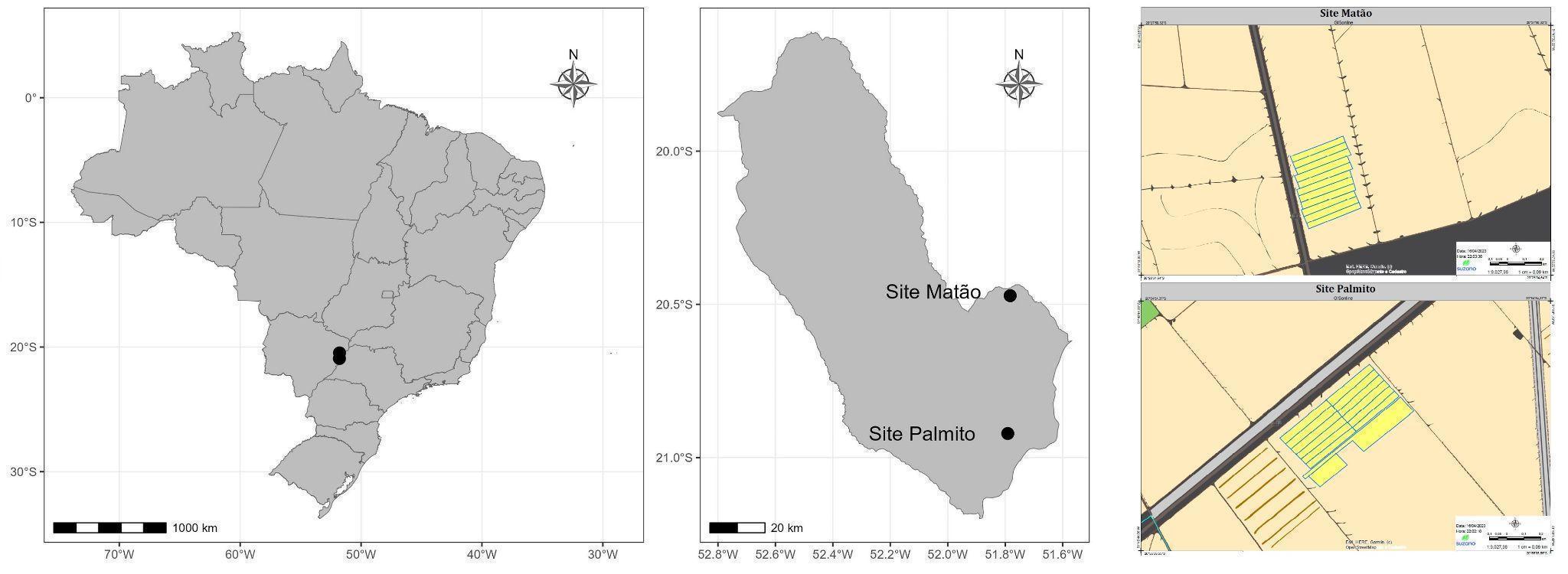
**

**Figure S1.** Cloned progeny trials of *Eucalyptus* spp., state of Mato Grosso do Sul, Brazil. MAT **(A)** and PAL **(B)** sites.

**Table S1.** Mean variance components and mean standard errors by fitting a single stage GBLUP-AD model in a multi-environmental trial (two sites: MAT and PAL) for one to five ramets/progeny.

| Number of ramets/progeny | Variance component | Estimate | Standard Error |
| --- | --- | --- | --- |
| 1 | $\sigma_{b_{s}}^{2}$ | 46.93 | 8.91 |
|  | $\sigma_{a_{s}}^{2}$ | 157.40 | 28.68 |
|  | $\sigma_{d_{s}}^{2}$ | 220.86 | 26.31 |
|  | $\sigma_{{e1}_{s}}^{2}$ | 761.60 | 25.15 |
|  | $\sigma_{{e1}_{s}}^{2}$ | 1218.42 | 35.86 |
| 2 | $\sigma_{b_{s}}^{2}$ | 48.68 | 6.04 |
|  | $\sigma_{a_{s}}^{2}$ | 212.44 | 29.76 |
|  | $\sigma_{d_{s}}^{2}$ | 301.43 | 22.98 |
|  | $\sigma_{{e1}_{s}}^{2}$ | 719.47 | 15.74 |
|  | $\sigma_{{e1}_{s}}^{2}$ | 1121.76 | 22.75 |
| 3 | $\sigma_{b_{s}}^{2}$ | 49.93 | 4.86 |
|  | $\sigma_{a_{s}}^{2}$ | 240.17 | 30.36 |
|  | $\sigma_{d_{s}}^{2}$ | 340.77 | 22.03 |
|  | $\sigma_{{e1}_{s}}^{2}$ | 709.42 | 12.28 |
|  | $\sigma_{{e1}_{s}}^{2}$ | 1082.30 | 17.58 |
| 4 | $\sigma_{b_{s}}^{2}$ | 50.74 | 4.17 |
|  | $\sigma_{a_{s}}^{2}$ | 257.60 | 30.79 |
|  | $\sigma_{d_{s}}^{2}$ | 365.16 | 21.63 |
|  | $\sigma_{{e1}_{s}}^{2}$ | 705.78 | 10.40 |
|  | $\sigma_{{e1}_{s}}^{2}$ | 1061.24 | 14.74 |
| 5 | $\sigma_{b_{s}}^{2}$ | 51.30 | 3.71 |
|  | $\sigma_{a_{s}}^{2}$ | 269.79 | 31.13 |
|  | $\sigma_{d_{s}}^{2}$ | 381.99 | 21.43 |
|  | $\sigma_{{e1}_{s}}^{2}$ | 704.14 | 9.18 |
|  | $\sigma_{{e1}_{s}}^{2}$ | 1048.33 | 12.91 |

Where: $\sigma_{b_{s}}^{2}$ is the incomplete block variance, $\sigma_{a_{s}}^{2}$ is the additive genetic variance, $\sigma_{d_{s}}^{2}$ is the dominance genetic variance, $\sigma_{{e1}_{s}}^{2}$ is the residual variance for site 1 (MAT), and $\sigma_{{e2}_{s}}^{2}$ is the residual variance for site 2 (PAL).
